# Differences in knowledge, attitude, and practice regarding hypertension by access to a community-based screening program (POSBINDU): A cross-sectional study from four districts in Indonesia

**DOI:** 10.1371/journal.pone.0303503

**Published:** 2024-05-14

**Authors:** Yusuf Ari Mashuri, Vitri Widyaningsih, Alimah Premanawasti, Jaap Koot, Zinzi Pardoel, Jeanet Landsman-Dijkstra, Maarten Postma, Ari Probandari

**Affiliations:** 1 Faculty of Medicine, Universitas Sebelas Maret, Surakarta, Indonesia; 2 Disease Control Research Group, Faculty of Medicine, Universitas Sebelas Maret, Surakarta, Indonesia; 3 Department of Health Sciences, University Medical Center Groningen, University of Groningen, Groningen, The Netherlands; University of Health Sciences Lahore, PAKISTAN

## Abstract

**Background:**

A high prevalence of hypertension is found in Low- and Middle-income Countries (LMICs) including in Indonesia. However, hypertension awareness, treatment, and control are relativity poor. A community-based program to screen and educate people on non-communicable disease prevention (POSBINDU) was launched by the Indonesian government. However, the association between participation in the POSBINDU program with increasing knowledge, attitude, and practice of hypertension has not been widely assessed. In this study, we compared the knowledge, attitudes, and practices among people who accessed the POSBINDU and those who did not access the POSBINDU program. Subsequently, factors associated with the knowledge, attitudes, and practices among people who accessed the POSBINDU and those who did not access the POSBINDU were explored.

**Methods:**

This was an observational study with a cross-sectional design measuring the knowledge, attitudes, and practices for hypertension control in four districts in Indonesia from October 2019 to January 2020. A total of 1,988 respondents were included in this study. A questionnaire was used to assess the knowledge, attitudes, and practices of hypertension. Simple logistic regression was used to investigate the correlation between the characteristics of respondents and knowledge, attitudes, and practice status. Multiple logistic regression tests were conducted to investigate factors associated with knowledge, attitudes, and practice status.

**Results:**

We found that people who accessed POSBINDU had higher odds of having better knowledge (aOR:1.4; 95%CI:1.2–1.8), however, accessed to POSBINDU was associated with lower attitudes (aOR:0.6; 85%CI: 0.5–0.7) and had no association with hypertension-related practice.

**Conclusion:**

People who accessed POSBINDU have an association with good knowledge, but the association with good attitude and practice was less clear. Therefore, an improvement in the POSBINDU program is needed to increase the attitudes and practices of hypertension.

## 1. Introduction

Non-communicable diseases (NCDs) prevalence keeps increasing both in high-income and low-middle-income countries (LMICs) [1]. Hypertension, one of the predominant NCDs, is highly prevalent in low-income and middle-income countries, including Indonesia [2]. The global burden of hypertension is estimated to cause 7.5 million deaths and contributed to 57 million Disability-Adjusted Life-Years (DALYs) or 3.7% of total DALYs [3].

The problem associated with the high prevalence of hypertension in LMICs is further complexified by the relatively poor hypertension awareness, treatment, and control in these countries [4, 5]. Moreover, in LMICs, significant socio-economic disparities in the awareness, treatment, and control of hypertension were observed [5]. In 2014, the Indonesian Family Life Survey (IFLS) showed that only 41.8% of individuals with hypertension were aware of their condition [6], and only 6.6% of respondents received treatment [7].

To improve awareness, screening uptakes, and linkage to care for NCDs, the government of Indonesia has launched an integrated program for NCDs. It includes a community-based program implemented in an integrated, routine, and periodic approach for the early detection and prevention of NCDs named Pos Pembinaan Terpadu (POSBINDU) [8]. POSBINDU explicitly comprises the prevention and early detection of NCDs particularly hypertension and diabetes, including counseling, health education, physical activities, and screening for NCDs and their risk factors, using anthropometric and blood pressure measurements [9]. The POSBINDU targets individuals aged 15 years and above, held by trained volunteers or cadres, and under the supervision of a Primary Health Centre (PHC) officer [8]. The POSBINDU aimed to improve attendants’ knowledge about hypertension [10], supporting their attitude and practice for preventing and controlling hypertension [11]. The POSBINDU attendants with hypertension in need of treatment are referred to Primary Health Care (PHC) to receive further diagnosis and medication. Additionally, hypertensive patients with complications, if necessary, will be referred to the hospital [7]. A recent study shows that hypertensive attendants of POSBINDU have higher odds of being treated [12] and are mostly aware of their condition [7, 12].

Hypertension knowledge, attitude, and practice are significantly associated with hypertension awareness. People who know about hypertension have a higher awareness of needing lifelong treatment [13, 14]. Good knowledge, attitudes, and practices of hypertension are recommended for hypertension management [15], such as diet, physical activity, and salt intake control [16]. So far, in Indonesia, the effectiveness of community activities in increasing knowledge, attitudes, and practices of hypertension has not been widely assessed. There are still few studies that compare the knowledge, attitudes, and practices of hypertension between people who accessed the POSBINDU and those who did not access the POSBINDU.

To fill this evidence gap, we aim to investigate the knowledge, attitudes, and practices among people who accessed the POSBINDU and those who did not access the POSBINDU. Subsequently, we also investigate factors associated with the knowledge, attitudes, and practices among people who accessed the POSBINDU and those who did not access the POSBINDU. Information on differences in knowledge, attitudes, and practices regarding hypertension between people accessing POSBINDU and those who do not access POSBINDU can provide insights into how to improve the community-based activities for screening and prevention of hypertension, particularly, in Indonesia and potentially other LMICs.

## 2. Material and methods

### 2.1. Study settings

This study was conducted in Kediri city and Jember district in the East Java province and Medan city and Deli Serdang district in the North Sumatra province from 1 October 2019 to 31 January 2020. The two provinces have a high burden of hypertension and were among the prioritized provinces for NCDs prevention and control in Indonesia.

Kediri city is an urban area while Jember district is a rural area in East Java. Kediri city consists of three sub-districts with a total population of 292,363. Most of the population is working in the industrial sector. In total, there are nine Primary Health Care (PHC) and nine POSBINDU sites in this city. The prevalence of hypertension in Kediri was 36.2% in 2019. The Jember district had a population of 2,538,921 residents and had 21 sub-districts where most of the population worked as farmers [17]. In total, there were 50 PHCs and 283 POSBINDU sites in Jember [18]. In 2019, the hypertension prevalence in the Jember district was 39.2% [17].

Medan is an urban area in North Sumatra, with 21 urban sub-districts with a total population of 2,524,341 [19]. Most of the population work in the manufacturing and trading industries. There are 33 PHCs and 124 POSBINDU sites and around 25.2% of adult individuals had hypertension in Medan [17]. Deli Serdang is a rural area with 1,931,411 residents and consists of twelve sub-districts and most of the population work as farmers [20]. There are 17 PHCs and 93 POSBINDU sites and around 31.4% of Deli Serdang residents were hypertensive. In total, we included 89 POSBINDU from 29 PHCs in Medan, 17 PHCs in Deli Serdang, 36 PHCs in Jember district, and 9 PHCs in Kediri city.

### 2.2. Study design

This was an observational study with a cross-sectional design to measure the knowledge, attitude, and practice regarding hypertension in the same period in different sites.

### 2.3. Sample size and sampling procedure

Multistage sampling was conducted to recruit the respondents. Based on the results of discussions with the Indonesian Ministry of Health, the provinces were selected purposively based on the NCDs burden and representing Java and outside Java conditions. Within each district, we further chose two districts representing more urban and rural areas, both with a high burden of NCDs. For each district, we randomly selected at least half of the PHCs. Within each PHCs we randomly select POSBINDU. Data of community members accessing POSBINDU were collected from the participants of POSBINDU within the PHC community, while data of community members who do not access POSBINDU were obtained from convenient sample from the PHC community. Districts with fewer participants were selected for all nine PHCs as the sample in Kediri due to the limited availability of participants in the area. All of the respondents were adults above 15 years old. In total, we recruited 2,011 respondents who filled out the questionnaire. Twenty-three (1.1%) respondents did not fill in the questionnaire completely, therefore the final data analyzed were from 1,988 (98.9%) respondents.

### 2.4. Instruments for data collection

The questionnaire was paper-based and developed based on previously used questionnaires [21], which we modified and performed backward-forward translation to Bahasa Indonesia. The questionnaire was piloted on 30 community attendants in Central Java province, with similar characteristics to the study respondents. Later, the questionnaire was revised to improve validity and reliability. The first part of the validated questionnaire was the sociodemographic and clinical condition questions. The second part of the questionnaire comprised nine knowledge questions, six attitude questions, and thirteen practice questions. The knowledge part covered the definition of the disease, signs and symptoms, risk factors, and complications of hypertension. The attitude questions focused on prevention and treatment, while the practice of a healthy diet, exercise, medicine consumption, and smoking behavior were covered in practice questions. Each question of the knowledge, attitude, and practice part of the questionnaire is included in Supplementary Material.

### 2.5. Ethical consideration

Ethical approval was obtained from the Medical and Health Research Ethics Committee (MHREC) Faculty of Medicine, Public Health, and Nursing, Universitas Gadjah Mada (KE/FK/0648/EC/2019). Written consent was obtained from all of the respondents. The informed consent form was signed before data collection.

### 2.6. Measurement of variables

Outcome variables are knowledge, attitudes, and practice scores on hypertension, exploring what was known and what was done with health care about hypertension that may reveal as the baseline information about misconceptions or misbehaviors about attitude and practice of hypertension [22]. We included sociodemographic variables: sex (male/female), age group (15–24, 25–44, 45–59, >60 years), education level (≤ primary school, secondary school, diploma/bachelor), occupation (working/not working), marital status (married/not married) and living area (rural/urban). Not working in the occupation variable means not having a job or being retired.

We included clinical characteristics such as current illness defined as respondents who were aware that they were diagnosed with hypertension. Respondents with hypertension were also asked about the medicine they were taking and whether they get their blood pressure checked regularly (once every week, more than 1 week, or never). The frequency of doctor’s visits was also asked in this study: once in a week or more than once per week.

### 2.7. Statistical analysis

Characteristics of respondents and the results of hypertension-related knowledge, attitudes and practices were presented descriptively using chi-square test to determine if there is a significant association between two categorical variables. For each question in the knowledge, attitude, and practice part, there were two options: yes or no or true or false. Each answer was scored one for the right answer, and zero for the wrong response. The total score of one participant varied from 0 to 32 (knowledge), from 0 to 12 (attitude), and 0 to 32 (practice). Median scores of knowledges, attitude, and practice (12, 4, 4 respectively) were used as the cut-off to determine the status of the respondents’ knowledge, attitude, and practice, in line with several KAP studies [23, 24]. This cut-off point would dichotomize the KAP scores into “good” and “poor” (for knowledge and practice scores) or “positive” and “negative” (for attitude scores). Multiple logistic regression tests were carried out to investigate factors associated with knowledge, attitudes, and practice status. STATA 14.0 software [25] was used to analyze the data. P-value <0.05 was considered significant.

### 2.8. Patient and public involvement

Patients or the public were not directly involved in the design, or conduct, or reporting, or dissemination plans of our research.

## 3. Results and discussion

### 3.1. Characteristics of the study respondents

Most respondents are female both the people who access POSBINDU and those who do not access it (85.1% vs. 73.2%). People who access POSBINDU are predominantly aged 45–59 years old and those who do not access POSBINDU are predominantly aged 25–44 years old (47.0% vs. 38.1%). Table 1 shows the detailed socio-demographic characteristics of the respondents.

**Table 1:** Socio-demographic characteristics of the respondents.

Characteristics	Access POSBINDU (1,115; 56%)	Do not access POSBINDU (873; 44%)	Total (1,988; 100%)	p-value
**Sex**				<0.001*
Female	949 (85.1)	639 (73.2)	1,588 (79.9)	
Male	166 (14.9)	234 (26.8)	400 (20.1)	
Missing	0	0	0	
**Age Group**				<0.001*
15–24	54 (4.8)	106 (12.1)	160 (8.1)	
25–44	333 (29.9)	333 (38.1)	666 (33.5)	
45–59	524 (47.0)	340 (39.0)	864 (43.5)	
>60	195 (17.5)	90 (10.3)	285 (14.3)	
Missing	9 (0.8)	4 (0.5)	13 (0.7)	
**Education**				0.001*
≤ Primary school	415 (37.2)	263 (30.1)	678 (34.1)	
Secondary School	617 (55.3)	512 (58.7)	1,129 (56.8)	
Diploma/Bachelor	80 (7.2)	92 (10.5)	172 (8.7)	
Missing	3 (0.3)	6 (0.7)	9 (0.4)	
**Occupation**				<0.001*
Working	330 (29.6)	384 (44.0)	714 (35.9)	
Not working	711 (63.8)	417 (47.8)	1,128 (56.7)	
Not reported	74 (6.6)	72 (8.3)	146 (7.3)	
**Marital Status**			1,988 (100)	<0.001*
Married	987 (88.5)	703 (80.5)	1,690 (85.0)	
Not married	121 (10.9)	159 (18.2)	280 (14.1)	
Missing	7 (0.6)	11 (1.3)	18 (0.9)	
**Area**				0.745
Urban	683 (61.3)	541 (62.0)	1,224 (61.6)	
Rural	432 (38.7)	332 (38.0)	8.4)	

*Statistically significant (p-value < 0.05)

Most people who access POSBINDU and those who do not access it are secondary school graduates (55.3% vs. 58.7%, respectively). Moreover, most of the people who access POSBINDU and those who do not access it were not employed (63.8% vs. 47.8%). Predominantly people who access POSBINDU and those who do not access it are married (88.5% vs. 80.5%).

### 3.2. Clinical-characteristic of the respondents

A quarter of all respondents (509; 25.6%) are hypertensive, with a higher proportion of hypertension among people who access POSBINDU compared to those who do not access it (30.6% vs. 19.2%). A higher proportion of hypertensive patients were getting treated among people who access POSBINDU compared to those who do not access (83.9% vs. 75.6%). Of the hypertensive respondents, 11.4% of them never checked their blood pressure regularly. The respondents who are currently under treatment for hypertension (81.1%) reported purchasing medicine in pharmacies. Furthermore, among the respondents who are currently under treatment for hypertension, 33.2% never visit the doctor. Detailed clinical characteristics of the respondents are shown in Table 2.

**Table 2:** Clinical characteristics of the respondents.

Characteristics	Access POSBINDU (1,115; 56%)	Do not access POSBINDU (873; 44%)	Total (1,988; 100%)	p-value
**Current illness**			1,988 (100)	<0.001*
Yes	341 (30.6)	168 (19.2)	509 (25.6)	
No	648 (58.1)	583(66.8)	1,231 (61.9)	
Missing	126 (11.3)	122 (14.0)	248 (12.5)	
**Blood Pressure Check** **			509 (100)	0.006*
Never	37 (10.9)	21 (12.5)	58 (11.4)	
1 week	240 (70.4)	94 (56.0)	334 (65.6)	
>1 week	27 (7.9)	19 (11.3)	46 (9.0)	
Missing	37 (10.9)	34 (20.2)	71 (14.0)	
**Current Treatment** **			509 (100)	0.075
Yes	286 (83.9)	127 (75.6)	413 (81.1)	
No	52 (15.3)	38 (22.6)	90 (17.7)	
Missing	3 (0.9)	3 (1.8)	6 (1.2)	
**Doctor visit** ***			413 (100)	0.325
Never	91 (31.8)	46 (36.2)	137 (33.2)	
1 week	159 (55.6)	68 (53.5)	227 (55.0)	
>1 week	32 (11.2)	9 (7.1)	41 (9.9)	
Missing	4 (1.4)	4 (3.2)	8 (1.9)	
**Medicine** ***			413 (100)	0.177
Amlodipine	155 (54.2)	64 (50.4)	219 (53.0)	
Captopril	102 (35.7)	51 (40.2)	153 (37.1)	
Bisoprolol	1 (0.4)	0 (0.0)	1 (0.2)	
Other	6 (2.1)	7 (5.5)	13 (3.2)	
Missing	22 (7.7)	5 (3.9)	27 (6.5)	
**Family history**			2,011 (100)	0.009*
Yes	157 (14.1)	88 (10.1)	245 (12.3)	
No	530 (47.5)	406 (46.5)	936 (47.1)	
Missing	428 (38.4)	379 (43.4)	(40.6)	

*statistically significant (p-value < 0.05)

**respondents who answered current illness ‘yes’

***respondents who answered current treatment ‘yes’

### 3.3. Knowledge of hypertension

In this study, we measured knowledge related to risk factors, symptoms, complications, and prognosis of hypertension. We found that for most questions, the proportion of correct answers for knowledge questions were observed among people who accessed POSBINDU (Table 1). However, for several aspects, there was a lower proportion of knowledge on hypertension risk factors among people who access POSBINDU compared to people who do not access it, particularly on smoking as a risk factor for hypertension (63.2 vs. 69.8%, respectively), heart failure as complication for hypertension (61.1% vs 68.7%, respectively), and that routine examination is needed for hypertension (88.0% vs 91.0%, respectively).

Regarding hypertension risk factors, people who access POSBINDU have a higher score compared to those who do not access POSBINDU related to hypertension risk factors particularly for poor diet (83.7% vs. 80.0%) and for obesity (77.3% vs. 73.7%). Meanwhile for symptoms, the common symptoms of hypertension known by the respondents included headaches (92.6%) with an almost equal proportion among people who access POSBINDU compared to those who do not access it (93.0% vs 92.0%), pain in the back of the neck (88.4% vs 85.7%), and blurry vision (75.1% vs 78.6%) (Table 3).

**Table 3:** Respondents’ knowledge, attitude, and practice of hypertension.

Variables	Accessing POSBINDU (1,115; 56%)	Not accessing POSBINDU (873; 44%)	Total (1,988; 100%)	p-value
**Knowledge**				
Definition of hypertension	1,020 (91.5)	781 (89.5)	1,801 (90.6)	0.310
Hypertension is a hereditary disease	733 (65.7)	519 (59.4)	1,252 (63.0)	0.013
People of all ages and genders can get hypertension	976 (87.5)	655 (75.0)	1,631 (82.0)	<0.001*
Risk factor for hypertension: family history	563 (50.5)	311 (35.6)	874 (44.0)	<0.001*
Risk factor for hypertension: obesity	862 (77.3)	643 (73.7)	1,505 (75.7)	0.136
Risk factors for hypertension: diet	933 (83.7)	698 (80.0)	1,631 (82.0)	0.100
Risk factor for hypertension: smoking	704 (63.1)	609 (69.8)	1,313 (66.1)	0.003*
Risk factor for hypertension: lack of physical exercise	599 (53.7)	442 (50.6)	1,041 (52.4)	0.000*
The main symptom of hypertension: headache	1,037 (93.0)	803 (92.0)	1,840 (92.6)	0.674
The main symptom of hypertension: pain on the back of the neck	986 (88.4)	748 (85.7)	1,734 (87.2)	0.129
Hypertension complication: blurry vision	837 (75.1)	686 (78.6)	1,523 (76.6)	0.183
Hypertension complications: heart failure	681 (61.1)	600 (68.7)	1,281 (64.4)	<0.001*
Hypertension complications: renal disease	731 (65.6)	494 (56.6)	1,225 (61.6)	<0.001*
Routine examination for hypertension is a blood pressure measurement	981 (88.0)	828 (94.9)	1,809 (91.0)	<0.001*
Hypertension can be cured	815 (73.1)	647 (74.1)	1,462 (73.5)	0.268
Hypertension treatment is a lifetime treatment	760 (68.2)	525 (60.1)	1,285 (64.6)	<0.001*
**Attitude**				
I do routine exams and measure vitals regularly if I get hypertension	1,044 (93.6)	818 (93.7)	1,862 (93.7)	0.304
If I have hypertension symptoms, I will go to the nearest health facility	1,065 (95.4)	820 (93.9)	1,885 (94.8)	0.113
I will cut down on salty and fatty foods to prevent hypertension	1,043 (93.5)	815 (93.4)	1,858 (93.5)	0.656
I will gradually lose weight to prevent hypertension	716 (64.2)	570 (65.3)	1,286 (64.7)	0.414
If I take medicine regularly, I don’t need to implement a healthy lifestyle	603 (54.1)	357 (40.9)	960 (48.3)	<0.001*
I will make lifestyle changes because it is the key element to preventing the worsening of hypertension	991 (88.9)	802 (91.9)	1,793 (90.2)	0.084*
**Practice**				
Healthy diet: the right amount	948 (85.0)	731 (83.7)	1,679 (84.5)	0.509
Healthy diet: right variety (T-plate model)	798 (71.6)	643 (73.7)	1,441 (72.5)	0.141
Healthy diet: right time (3 main meals, 2–3 light snacks)	805 (72.2)	645 (73.9)	1,450 (72.9)	0.135
I have implemented this diet to reduce hypertension risk	577 (51.8)	364 (41.7)	941 (47.3)	<0.001*
I will implement this diet if I am diagnosed with hypertension	725 (65.0)	570 (65.3)	1,295 (65.1)	0.991
I do not need regular exercise because I’m not diagnosed with hypertension	380 (34.1)	283 (32.4)	663 (33.4)	0.329
I exercise regularly, 3 times a week for 30 minutes	359 (32.2)	348 (39.9)	707 (35.6)	<0.001*
I prefer irregular strenuous exercise rather than regular light exercise	335 (30.0)	197 (22.6)	532 (26.8)	<0.001*
I do not need to exercise because I have lots of house chores	423 (37.9)	391 (44.8)	814 (41.0)	0.008*
I prefer herbal medicine rather than chemical medicine	463 (41.5)	430 (49.3)	893 (44.9)	<0.001*
I smoke	97 (8.7)	166 (19.0)	263 (13.2)	<0.001*
If yes, I intend to quit in one year**	167 (15.0)	233 (26.7)	400 (20.1)	<0.001*
I will quit smoking if I am diagnosed with hypertension**	232 (20.8)	275 (31.5)	507 (25.5)	<0.001*
I will go to the doctor regularly if I am diagnosed with hypertension	925 (83.0)	738 (84.5)	1,663 (83.7)	0.350
I will help give motivation and education to other hypertension patients	1,047 (93.9)	818 (93.7)	1,865 (93.8)	0.500

*statistically significant (p-value < 0.05)

** Respondents who answered I smoke ‘yes’

On the other hand, less than two-thirds of respondents knew that heart failure and renal disease are complications of hypertension for people who access POSBINDU and those who do not access it (64.4% and 61.6%) with non-significant differences between people who access POSBINDU versus people who do not access it (61.1% vs. 68.8%; 65.6% vs 56.7%). Regarding prognosis of hypertension, only three-quarters of the respondents knew that hypertension could not be cured (73.1% vs. 74.1%) and only two-thirds understand that the treatment is for a lifetime with a significantly lower portion among people who do not access POSBINDU versus those access it (60.1% vs. 68.1%) (Table 3).

### 3.4. Attitude on hypertension

Almost all the respondents agree that they have to visit a health facility when they have hypertension symptoms for POSBINDU (94.8%), with no significant different between people who access POSBINDU vs those who do not access POSBINDU) (95.4% vs 93.9% respectively). However, almost half of the respondents answered that a healthy lifestyle is not important when they already take medicine regularly, with a significant difference between people who access POSBINDU (54.1%) compared to people who do not access POSBINDU (40.9%).

### 3.5. Hypertension control-related practice

From 15 hypertension control-related practice questions, we found there were significant differences in seven practices between people who access POSBINDU and those who do not access POSBINDU. People who access POSBINDU reported a higher proportion of healthy diet (51.8%) and preferring a regular strenuous exercise compared to light exercise (30.0%), compared to only 41.7% and 22.6% in people who do not access POSBINDU. A lower proportion of smoking was also reported among people who access POSBINDU (8.7%) compared to people who do not access POSBINDU (19.0%). However, only a quarter of respondents (25.5%) answered “intending to quit smoking when diagnosed with hypertension.” (Table 3). There was slightly lower proportion of respondents reporting to exercise three times for 30 minutes/week among people who access POSBINDU compared to those who do not access POSBINDU (32.2% vs 39.9%, respectively).

### 3.6. Factors associated with knowledge, attitude, and practice on hypertension

In this study, we found that education status, working status, and POSBINDU access were significantly associated with knowledge of hypertension (Table 4). Respondents having lower education status at primary school (aOR:0.4; 95%CI:0.3–0.6), working (aOR:0.7; 95%CI:0.6–0.9), who did not have hypertension (aOR:0.6; 95%CI:0.5–0.7) had higher odds having lower knowledge on hypertension. Meanwhile, people who access POSBINDU had higher odds of having better knowledge of hypertension compared to people who do not access it (aOR:1.4; 95%CI:1.2–1.8).

**Table 4:** Factors associated with knowledge, attitude, and practice on hypertension.

Variables	Knowledge		Attitude		Practice	
	OR (95% CI)	aOR^+^ (95% CI)	OR (95% CI)	aOR^+^ (95% CI)	OR (95% CI)	aOR^+^ (95% CI)
**Sex**						
Female	1.1 (0.8–1.4)	1. 3 (1.0–1.8)	1.1 (0.8–1.5)	1.1 (0.8–1.6)	1.3 (1.0–1.8)	1.4 (1.0–1.9)
Male	1	1	1	1	1	1
**Age Group**						
15–24	1.0 (0.6–1.6)	1.2 (0.7–2.0)	2.1 (1.2–3.5)*	2.0 (1.2–3.4)*	1.5 (0.9–2.5)	1.5 (0.8–2.6)
25–44	1.0 (0.7–1.7)	1.1 (0.9–1.5)	1.1 (0.9–1.4)	1.1 (0.8–1.4)	1.0 (0.8–1.2)	1.0 (0.8–1.3
45–59	1	1	1	1	1	1
>60	1.2 (0.9–1.7)	1.3 (0.9–1.9)	1.0 (0.5–1.2)	1.0 (0.7–1.3)	1.5 (1.1–2.1)*	1.4 (1.0–2.1)
**Education**						
Primary school	0.4 (0.2–0.6)*	0.4 (0.3–0.6)*	0.6 (0.4–0.9)*	0.6 (0.4–0.9)*	0.5 (0.3–0.8)*	0.6 (0.4–0.8)*
Secondary School	0.5 (0.3–0.9)*	0.6 (0.4–0.8)*	0.8 (0.5–1.2)	0.8 (0.5–1.2)	0.7 (0.6–1.0)*	0.7 (0.5–1.0)
Diploma/Bachelor	1	1	1	1	1	1
**Occupation**						
Working	0.7 (0.4–1.0)*	0.7 (0.6–0.9)*	1.1 (0.8–1.4)	1.2 (0.9–1.4)	0.9 (0.7–1.1)	0.8 (0.7–1.0)
Did not working	1	1	1	1	1	1
Not reported	0.7 (0.4–1.1)	0.7 (0.4–1.1)	1.3 (0.8–2.1)	2.2 (1.9–2.4)	1.2 (0.7–2.0)	1.9 (1.7–2.2)
**Marital status**						
Married	1.1 (0.8–1.6)	1.2 (0.8–1.7)	1.8 (1.3–2.6)*	1.8 (1.3–2.5)*	1.1 (0.7–1.5)	1.0 (0.7–1.5)
Not married	1	1	1	1	1	1
**Current illness**						
No	0.6 (0.5–0.7)*	0.6 (0.5–0.7)*	1.4 (1.1–1.7)*	1.4 (1.1–1.7)*	0.5 (0.4–0.7)*	0.5 (0.4–0.6)*
Yes	1	1	1	1	1	1
**POSBINDU access**						
Yes	1.4 (1.1–1.7)*	1.4 (1.2–1.8)*	0.6 (0.5–0.7)*	0.6 (0.5–0.7)*	0.9 (0.8–1.2)	0.9 (0.7–1.1)
No	1	1	1	1	1	1
**Area**						
Urban	0.9 (0.7–1.1)	0.8 (0.7–1.1)	0.5 (1.3–4.8)*	0.5 (0.4–0.6)*	0.7 (0.6–0.9)	0.7 (0.6–0.9)*
Rural	1	1	1	1	1	1

*statistically significant (p-value <0.05)

^+^aOR: adjusted for socio-demographic characteristics, clinical characteristics, and access to POSBINDU

The findings showed that younger respondents with age within 15–24 years old (aOR:2.0; 95%CI:1.2–3.4), married (aOR:1.8; 95%CI:1.3–2.5) and respondents who did not have hypertension (aOR:1.4; 95%CI:1.1–1.7) have higher odds of having a better attitude towards hypertension (Table 4). In contrast, respondents with primary school education (aOR:0.6; 95%CI:0.4–0.9), access POSBINDU (aOR:0.6; 85%CI: 0.5–0.7), and living in the urban area (aOR:0.5; 95%CI:0.4–0.6)) had lower odds of having a good attitude.

Furthermore, individuals having lower education levels (aOR:0.6; 95%CI:0.4–0.8), who did not have hypertension (aOR:0.5; 95%CI:0.4–0.6), and live in urban area (aOR:0.7; 95%CI:0.6–0.9) had lower odds of having good practice on hypertension.

## 4. Discussion

In this study, we have shown that in Indonesia, people accessing the community-based program POSBINDU were associated with having good knowledge of hypertension risk factors and complications. However, our findings revealed that people who accessed POSBINDU were associated with lower scores on attitudes and no association with practice related to hypertension.

### 4.1. Socio-demographic and clinical characteristics of respondents

We found that female and older populations have more access to POSBINDU. An explanation for this finding can be found in previous research, which revealed barriers to participation in the male and younger populations, such as inconvenient schedules and low awareness of hypertension screening [26]. We found that the people accessing POSBINDU were predominantly female and aged 45–59 years old. This finding relates to previous research in Indonesia which reported that the majority of POSBINDU attendants were female and belonged to the age group 45–59 years old [26, 27]. One of the main barriers to access in these groups can be the opening hours of POSBINDU, which mostly occurred during working hours [28].

This study showed that around one-fourth of respondents were found with high blood pressure. When compared to self-reported hypertension in the two provinces, North Sumatra (6.07%) and East Java (8.59%), the proportion in our research (25.6%) is higher than the one from previous national research [17]. This discrepancy might be due to differences in the measurement of hypertension. In this study, the respondents reported their hypertension diagnosis, while in the survey, the respondents were measured by a trained enumerator. The lower proportion might be due to lower awareness among the respondents. Another study in a low-income country, Kenya, showed that less than one-third of individuals with hypertension are aware of their condition and only a minority of them are being treated and having their hypertension controlled [29].

More than half of the hypertensive respondents visited the doctor for treatment but only two third of them took medication after being diagnosed. Our findings are in line with a study in Korea that showed only 13.2% of hypertensive respondents take prescribed medication [30]. This might be related to lacking access to treatment or financing barriers [5]. Another possible explanation could be that patients generally believe that their hypertension is not in a concerning stage, resulting in reduced acceptance of the diagnosis and a lower sense of urgency for high blood pressure treatment [31].

### 4.2. Knowledge, attitude, practice on hypertension among respondents

This study shows that most respondents understood the definition of hypertension, had routine blood pressure checks, and perceived that poor diet and obesity were risk factors for hypertension. Respondents also knew that headaches, pain in the back of the neck, and blurred vision are common symptoms of hypertension. However, many respondents did not understand that smoking, physical inactivity, and family history are also risk factors for hypertension, even though many studies show that these conditions are important factors in hypertension control [32–35]. Moreover, many respondents still do not know the complications of hypertension such as heart failure and kidney disease. This is not in accordance with previous research that there is an association between adherence to hypertension therapy and knowledge of hypertension complications [36].

Significant differences were found in the knowledge of risk factors for hypertension such as diet, smoking, and lack of physical exercise between people who accessed POSBINDU and those who did not access POSBINDU. Our findings are in line with research in Nepal and China, that health community attendants have better knowledge of hypertension than non-community attendants [37, 38]. Thus, this study suggests that improving hypertension risk control can be done by educating the community on hypertension risks including poor diet, smoking, and lack of physical exercise. Almost half of the respondents answered that maintaining a healthy lifestyle becomes less important when they are already on medication. This finding is in contrast with another study that most of the population prefers a healthy lifestyle rather than medication [39–41] Our findings align with another study’s results, which indicated that individuals diagnosed with lifestyle-related diseases often neglect their dietary and exercise habits. This behavior might come from their perception that medication therapy holds greater significance than diet and exercise that they may believe for relying on medications can make up for their unhealthy lifestyle choices [42].

We also found that people who accessed POSBINDU had higher practice scores regarding diet, regular physical exercise, and smoking behavior, compared to people who did not access POSBINDU. This confirms earlier findings, in which community members who practice healthy lifestyles can control their blood pressure and benefit in the long term [43].

We found that respondents with higher education, no working status, hypertension, and being people who accessed POSBINDU had higher odds of having a better knowledge of hypertension. This is in line with earlier research that educational background has an important positive influence on patients’ knowledge and beliefs about non-communicable diseases [44]. Also, the respondents who are not working have higher odds of having good knowledge which can be explained by the fact that retired-from-work individuals were also included in the “did not work” category. They might have more chances to learn about hypertension in their previous workplace and their retirement period [45]. One study also pointed out that individuals with employment have a low level of awareness of hypertension [12]. Respondents already diagnosed with hypertension may be exposed to information from healthcare providers or educational material during their visit [39]. This could explain why they have adequate knowledge about hypertension risk factors and complications [40].

We found that a good attitude is associated with age, where the younger age group at 15–24 years old has higher odds of having a good attitude compared to the older age group. This result is different from another study in that older individuals have a better attitude toward hypertension [46] as their age increases [47]. Respondents with no current illness have higher attitude scores, in contrast with another study that hypertensive patients tend to have a more favorable attitude towards lifestyle modification [48].

Our results showed that good practice is associated with current illness status. Hypertensive respondents had better practice scores because they could gather information from healthcare providers and physicians to improve their new behavior and practice [49]. They have good self-care practices such as good diet practice and non-smoking behavior [50]. Respondents living in rural areas also had higher odds of having good practice. This finding contrasts with another study that shows rural area communities tend to have lower education and strong beliefs in herbal and traditional medicines rather than medication, thus influencing their practices on hypertension [51].

On the other hand, access to POSBINDU had a significant influence on people’s knowledge of hypertension. Therefore, people who access POSBINDU have better knowledge of hypertension than those who do not access POSBINDU. This can be explained by another study which showed that people who access POSBINDU get health information related to NCDs, especially hypertension from health workers or cadres during POSBINDU visits [8]. This interactive education can increase their knowledge [47].

In this study, people who did not access POSBINDU had a better attitude and practice than who accessed POSBINDU. A possible explanation of this finding is that community-based healthcare interventions in Indonesia, such as POSBINDU focus on health education and screening for the risk of NCDs. The program successfully identified and educated people at risk [35] but has not adequately touched on the attitude and practice aspects. In contrast, another study shows knowledge has a significant relationship with attitude and practice [49, 52] Another factor that influences our result is respondent age. Our study shows that people who access POSBINDU are significantly older, and those aged 60 years or older have better practices regarding NCDs.

## 5. Conclusions

There are differences in knowledge, attitude, and practice regarding hypertension by access to a Community-based Screening Program (POSBINDU). Significant differences were found in the knowledge of hypertension risk, complications and routine examination, the attitude toward medication and a healthy lifestyle, also at the practice aspect on hypertension risk, regular exercise, and smoking behavior between people who access POSBINDU and those who do not access. People who access POSBINDU are associated with good knowledge but not with a good attitude or practice.

This study showed the need for further improvement of POSBINDU to increase the coverage (male and younger attendants) and to diversify activities. These activities include easier information access on the risk factors of hypertension and integration of physical activities during POSBINDU.

## 6. Strengths and limitations

We evaluated the knowledge, attitudes, and practices of people who access POSBINDU and those who do not access on a large-scale respondents from our cities and districts in Indonesia. Hence, we provide insights into the outcome of knowledge, attitudes, and practices of hypertension resulting from participation in POSBINDU as a community-based NCD screening.

However, this study also has limitations. First, our study only included four districts in Java and Sumatra islands; thus, the generalizability to the national-wide context should be taken cautiously. Second, we conducted a cross-sectional study that does not determine the causality or temporal relationship, so assessing the change in knowledge, attitudes, and practices due to access in POSBINDU is difficult.

## 7. Implications

Education in POSBINDU is needed, to maintain and improve knowledge regarding risk factors of hypertension. Increasing knowledge regarding hypertension, POSBINDU can use blood pressure action sheets which are considered easier to understand than leaflets [53]. Health education strategies that can be implemented are counseling sessions and interactive workshops, increasing self-care skills for blood pressure control action for the hypertensive patient [38]. Healthy lifestyle education and consultation can be improved and maximized by using various media and ensuring easy access to information and education in POSBINDU [41, 54]. Improving the attitude and practice aspect of regular physical exercise can be conducted by including physical programs such as aerobic training, or cooking sessions in the POSBINDU activities. Moreover, maximizing medication and lifestyle change strategies can also be implemented [38].

Enhancing knowledge and skills can be achieved through a comprehensive education program focused on health, effective communication training, equipment usage, and engaging various stakeholders. Such education contributes to higher community involvement, leading to positive changes in health behaviors, self-confidence in managing health, better health literacy, and reduced risk factors associated with non-communicable diseases (NCDs). Moreover, comprehensive community-based interventions, incorporating diverse strategies like physical exercise and fostering self-confidence, have proven to yield improved health outcomes [55].

## Abbreviations

DALYs: Disability-Adjusted Life-Years
GACD: Global Alliance for Chronic Diseases
LMIC: in Low-and-Middle Income Countries
MHREC: Medical and Health Research Ethics Committee
NCD: Noncommunicable Diseases
PHC: Primary Health Care
POSBINDU: Pos Pembinaan Terpadu
SUNI-SEA: Scaling-up NCD Intervention in South-East Asia
